# A Sequence Polymorphism in *MSTN* Predicts Sprinting Ability and Racing Stamina in Thoroughbred Horses

**DOI:** 10.1371/journal.pone.0008645

**Published:** 2010-01-20

**Authors:** Emmeline W. Hill, Jingjing Gu, Suzanne S. Eivers, Rita G. Fonseca, Beatrice A. McGivney, Preethi Govindarajan, Nick Orr, Lisa M. Katz, David MacHugh

**Affiliations:** 1 Animal Genomics Laboratory, School of Agriculture, Food Science and Veterinary Medicine, University College Dublin, Dublin, Ireland; 2 Conway Institute of Biomolecular and Biomedical Research, University College Dublin, Dublin, Ireland; University of Utah, United States of America

## Abstract

Variants of the *MSTN* gene encoding myostatin are associated with muscle hypertrophy phenotypes in a range of mammalian species, most notably cattle, dogs, mice, and humans. Using a sample of registered Thoroughbred horses (*n* = 148), we have identified a novel *MSTN* sequence polymorphism that is strongly associated (g.66493737C>T, *P* = 4.85×10^−8^) with best race distance among elite racehorses (*n* = 79). This observation was independently validated (*P* = 1.91×10^−6^) in a resampled group of Thoroughbreds (*n* = 62) and in a cohort of Thoroughbreds (*n* = 37, *P* = 0.0047) produced by the same trainer. We observed that C/C horses are suited to fast, short-distance races; C/T horses compete favorably in middle-distance races; and T/T horses have greater stamina. Evaluation of retrospective racecourse performance (*n* = 142) and stallion progeny performance predict that C/C and C/T horses are more likely to be successful two-year-old racehorses than T/T animals. Here we describe for the first time the identification of a gene variant in Thoroughbred racehorses that is predictive of genetic potential for an athletic phenotype.

## Introduction

Myostatin gene (*MSTN*) variants have previously been shown to contribute to muscle hypertrophy in a range of mammalian species [1], [2], [3], [4], [5]. In particular, whippet racing dogs that are heterozygote for a *MSTN* polymorphism have significantly greater racing ability than both homozygote wild-type dogs and homozygotes for the mutation that have an increased musculature that is detrimental to performance [1]. Horses, in particular Thoroughbreds, have a very high muscle mass to body weight ratio (55%) compared to other mammalian species (30–40%) [6] and the Thoroughbred genome contains evidence for selection for muscle strength phenotypes [7].

The Thoroughbred horse industry is a multi-billion dollar international enterprise engaged in the breeding, training and racing of elite racehorses. A Thoroughbred is a registered racehorse that can trace its ancestry to one of three foundation stallions and the approximately 30 foundation mares entered in The General Studbook, 1791 [8]. During the 300-year development of the breed racehorses have been intensely selected for athletic phenotypes that enable superior racecourse performance in particular types of races. There are two types of Thoroughbred race: National Hunt races are run over hurdles or steeplechase fences over distances of up to 4.5 miles (7,200 m), while Flat races have no obstacles and are run over distances ranging from five furlongs (5/8 mile or 1,006 m) to 20 furlongs (4,024 m). The International Federation of Horseracing Authorities recognizes five race distance categories: Sprint (5–6.5 f, ≤1,300 m), Mile (6.51–9.49 f, 1,301–1,900 m), Intermediate (9.5–10.5 f, 1,901–2,112 m), Long (10.51–13.5 f, 2,114–2,716 m) and Extended (>13.51 f, >2,717 m) races (International Federation of Horseracing Authorities Classifications, www.horseracingintfed.com) [Note: 1 furlong = 1/8 mile = 201.2 meters] and horses that compete in these races are generally termed ‘sprinters’ (<6 furlongs), ‘middle distance’ or ‘milers’ (7–8 f) or ‘stayers’ (>8 f). Similar to their human counterparts, sprint racing Thoroughbreds are generally more compact and muscular than horses suited to longer distance races.

To-date, no sequence variants have been reported in genomic *MSTN* sequence in Thoroughbred horses and no *MSTN* SNPs are documented in the EquCab2.0 SNP database. Therefore, we have investigated sequence variation in the equine *MSTN* gene, which contains three exons and spans 6,172 bp on chromosome 18 (reverse strand nt 66489608—66495780, EquCab2.0) [9] and investigated associations between *MSTN* sequence variants and racing phenotypes.

## Results and Discussion

Novel sequence variants were identified by re-sequencing the equine *MSTN* gene in 24 unrelated Thoroughbred horses using 13 overlapping primer pairs (Table S1) spanning all three exons and 288 bp of the 5′ upstream region. Although no exonic sequence variants were detected, six SNPs were detected in intron 1 of *MSTN* [nt 66492979—66494807] (Table S2).

To investigate associations between *MSTN* sequence variants and racing phenotypes we genotyped *n = *148 Thoroughbred horses. Four of the six *MSTN* sequence polymorphisms displayed MAF<0.05 in Thoroughbreds (Table S3) and were excluded from the association analyses. We performed a series of population-based case-control investigations by separating the Thoroughbreds on the basis of retrospective racecourse performance into discrete cohorts containing unrelated animals (Table S4). Individual genotypes at the two SNPs used for the analyses (g.66493737C>T and g.66494218A>C) were not more common among elite Group race winning Thoroughbreds (Thoroughbred-elite, TBE) than horses that had never won a race (Thoroughbred-other, TBO) [Table 1]. Also, no association was detected when handicap ratings, reflecting retrospective racing ability, were evaluated as a quantitative phenotype. However, considering the relative contribution of muscle power to sprint and longer distance racing we subdivided the elite Group race winning animals into those that had won their best (most valuable or highest grade) race over distances ≤8 f (furlongs, *n = *51) and those that had won their best race over distances >8 f (*n = *35) and found highly significant associations [Note: 1 furlong = 1/8 mile = 201.2 meters]. For all analyses the significance of association was consistently higher for g.66493737C>T than g.66494218A>C and the linkage disequilibrium between these SNPs was relatively high (*r* ^2^ = 0.50). Conditioning on each SNP using a logistic regression model identified an independent effect for g.66493737C>T on g.66494218A>C (*P* = 0.0108) but not for g.66494218A>C on g.66493737C>T (*P* = 0.7388) and therefore we considered further only the results for g.66493737C>T. Among the two distance cohorts we found a highly significant (*P* = 3.70×10^−5^) association with g.66493737C>T and this association became marginally stronger (*P = *1.88×10^−5^) when the short distance cohort was further subdivided into animals (*n* = 43) that had won their best race over distances ≤7 f (Table 1).

**Table 1:** Case-control association test results for a series of cohort comparisons for g.66493737C>T.

**Pop 1 ** ***vs*** ** Pop 2**	**Freq T_ Pop 1**	**Freq T_Pop 2**	***CHISQ***	***P***	***OR***
**TBE ** ***vs*** ** TBO**	0.443	0.425	0.09	0.764	-
**TBE>8 f ** ***vs*** ** TBE≤8 f**	0.641	0.309	17.02	3.70E-05	3.996
**TBE>8 f ** ***vs*** ** TBE≤7 f**	0.641	0.282	18.31	1.88E-05	4.538
**TBE>8 f ** ***vs*** ** TBO**	0.641	0.425	7.76	0.005	-
**TBE≤8 f ** ***vs*** ** TBO**	0.309	0.425	3.06	0.080	-
**TBE≤7 f ** ***vs*** ** TBO**	0.282	0.425	4.15	0.042	-
	**TBE>8 f**	**TBE≤7 f**	***P***		
**Genotypic (C/C, C/T, T/T)**	0/23/9	21/23/3	1.18E-06		
**Trend (C, T)**	23/41	65/29	5.23E-06		

TBE: elite Group race winning Thoroughbreds; TBO: other non-winning Thoroughbreds; TBE>8 f, TBE≤8 f and TBE≤7 f: elite Group race winning Thoroughbreds that won their best (most valuable or highest grade) races over distances>8 f, ≤8 f and ≤7 f. In each case the frequency of the g.66493737-T allele is given. Odds ratios were calculated for the two most significant results. Best-fit model results for genotypic and trend tests for g.66493737C>T association with elite Group race winning performance over distances ≤7 f are also shown.

The C allele was twice as frequent in the short distance (≤7 f) than in the long distance (>8 f) cohort (0.72 and 0.36 respectively) corresponding to an odds ratio of 4.54 (95% C.I. 2.23—9.23). When all Thoroughbreds were considered together the locus conformed to expected Hardy-Weinberg (HWE) proportions (Table S5). However, there was a significant (*P = *0.0018) deviation from HWE in the longer distance cohort, possibly due to selection at this locus; while the C/C genotype was the most common genotype among sprinters (≤7 f; 0.51), it was absent in the longer distance cohort. Genotype trend effects were modeled by estimating the risk associated with a linear trend in magnitude of effect relative to the common homozygote, heterozygote, and rare homozygote genotype using the Cochran-Armitage test for the trend model. The most parsimonious model was the genotypic model (*P* = 1.18×10^−6^) indicating that genotypes are predictive of optimum racing distance (Table 1).

**Figure 1:**
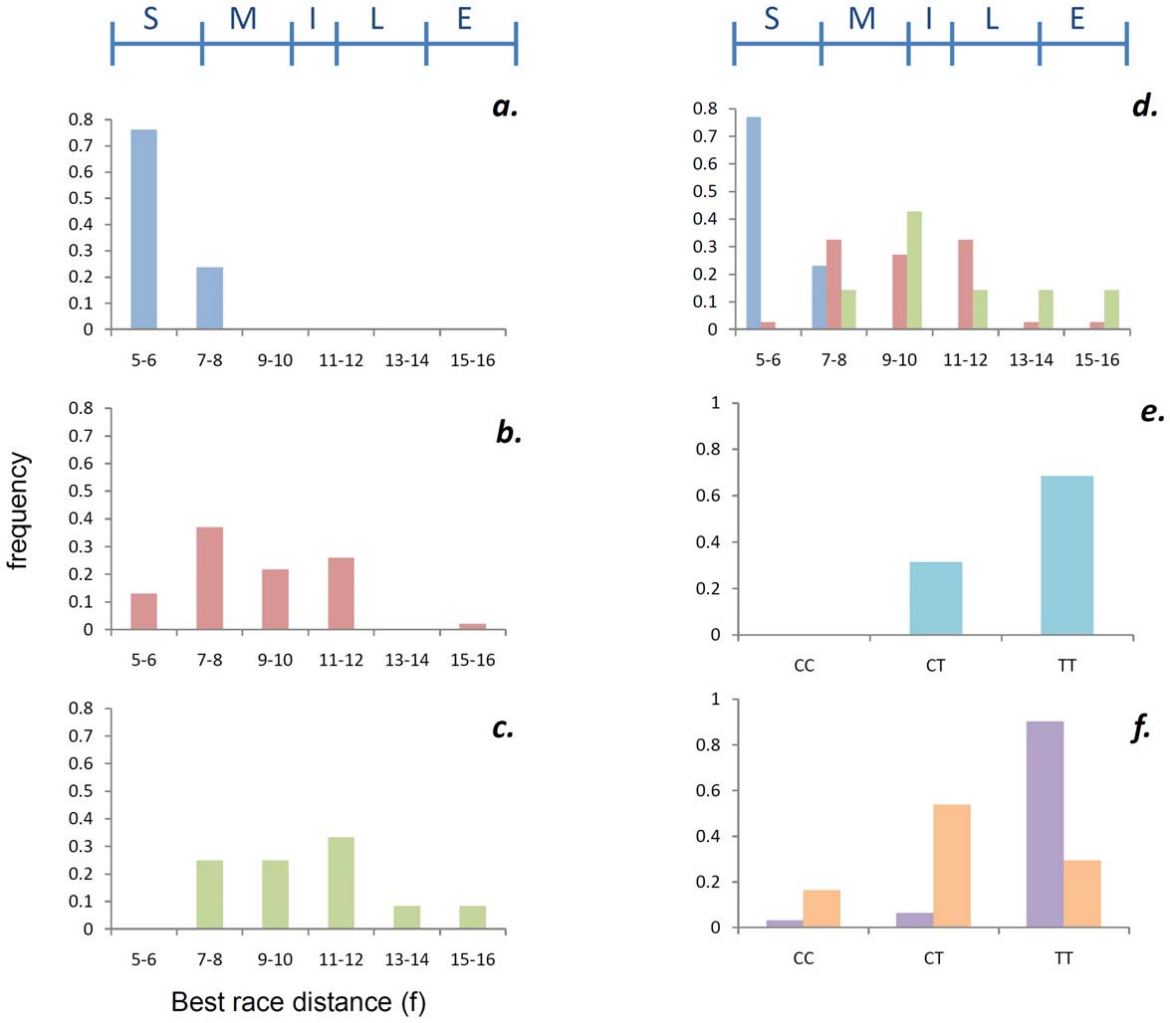
*MSTN* genotype distributions among Thoroughbred horses. Distribution of **a.** C/C, **b.** C/T and **c.** T/T genotypes among (*n* = 79) Group race winning Thoroughbreds. The non-uniformity of the distributions (**b.** and **c.**) may be explained by the absence of 9 f races among the 84 Group 1 races held in Great Britain. **d.** To avoid the bias introduced by racing distances for two-year-olds limited to ≤8 f (in Great Britain and Ireland), the distribution of genotypes was plotted for individuals with best race distance >8 f and for those two-year-olds that won their best race ≤8 f mean three-year-old race distances were used. **e.** Genotype distributions among National Hunt racing Thoroughbreds (aqua) **f.** Genotype distributions among Flat racing Thoroughbred (orange) and Egyptian (purple) horse populations. **a.–e.** C/C - blue, C/T - red, T/T - green, vertical axes indicate frequency, horizontal axes indicate best race distance (furlongs). The International Federation of Horseracing Authorities recognizes five race distance categories: Sprint (5–6.5 f, ≤1,300 m), Mile (6.51–9.49 f, 1,301–1,900 m), Intermediate (9.5–10.5 f, 1,901–2,112 m), Long (10.51–13.5 f, 2,114–2,716 m) and Extended (>13.51 f, >2,717 m); S-M-I-L-E [Note: 1 furlong = 1/8 mile = 201.2 meters].

**Figure 2:**
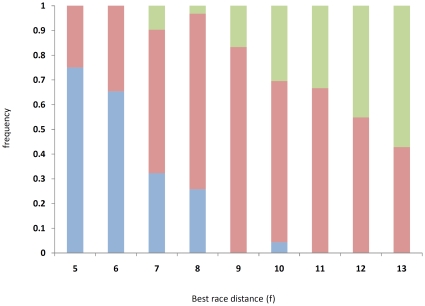
Optimal *MSTN* genotype for racing distance. Distribution of C/C (blue), C/T (red) and T/T (green) genotypes among *n* = 179 Group and Listed race winning Thoroughbreds.

Considering best race distance (BRD) as a quantitative trait, we analyzed the data for the elite cohort using the distance (furlongs) of the highest grade or most valuable Group race won as the phenotype (*n* = 79). BRD was highly significantly associated (*P = *4.85×10^−8^) with the g.66493737C>T SNP (Table 2). This result was independently validated (*P* = 1.91×10^−6^) in a re-sampled group of unrelated elite (Group and Listed race winners) Thoroughbreds (*n* = 62) and in a cohort of 37 elite racehorses (*P* = 0.0047) produced by the same trainer. For each genotype we determined the mean BRD in the original sample (Table 2): C/C mean = 6.2±0.8 f; C/T mean = 9.1±2.4 f; and T/T mean = 10.5±2.7 f. A distribution of the genotypes in two furlong increments is shown in Figure 1a–c. It is important to note that a bias may be introduced to these distances as two-year-old Group races are limited to ≤8 f in Ireland and Great Britain (there are only three Group races for two-year-olds in Europe>8 f). Therefore we replaced BRD for horses that had won their only Group race at two years old with the average distance of their three-year-old races (*n = *73), which resulted in a marginal increase in the means for the three genotypes (C/C mean = 6.4±1.0 f; C/T mean = 9.7±2.0 f; and T/T mean = 10.9±2.4 f) and an increase in the significance of association (*P = *5.45×10^−9^) (Figure 1d). A striking trend was observed when the genotypes for all 179 Group and Listed race winners (including relatives) were evaluated for BRD (Figure 2). As the distance of the races increased the frequency of the C/C genotype decreased and was almost absent at distances >8 f (*n* = 1 C/C sample had BRD = 10 f), while the frequency of the T/T genotype increased from approximately the same point.

**Table 2:** Quantitative trait association test results and best race distance (BRD) means for Association test sample; Association test sample using mean three-year-old BRD as phenotype (for two-year-olds that won their best race ≤8 f); Replication sample I; and Replication sample II.

	Quantitative association test results					Best race distance means			
*n*	*BETA*	*SE*	*R2*	*T*	*P*	GENO	C/C	C/T	T/T
79	2.308	0.381	0.322	6.052	4.85E-08	**COUNTS**	21	46	12
						**FREQ**	0.266	0.582	0.152
	**Association test sample**					**MEAN**	6.167	9.087	10.540
						**SD**	0.827	2.365	2.742
73	2.390	0.360	0.383	6.635	5.46E-09	**COUNTS**	19	42	12
						**FREQ**	0.260	0.575	0.164
	**Association test sample (3yo)**					**MEAN**	6.421	9.682	10.930
						**SD**	1.022	2.081	2.441
62	1.944	0.368	0.319	5.276	1.91E-06	**COUNTS**	17	34	11
						**FREQ**	0.274	0.548	0.177
	**Replication sample I**					**MEAN**	6.559	8.971	10.32
						**SD**	1.144	2.195	1.978
37	1.500	0.497	0.207	3.021	0.005	**COUNTS**	7	23	7
						**FREQ**	0.189	0.622	0.189
	**Replication sample II**					**MEAN**	6.714	8.217	9.714
						**SD**	1.704	1.930	1.890

Thirty-eight National Hunt (races over obstacles and distances 16–36 f) racehorses were also genotyped for the g.66493737C>T SNP. Remarkably, the C/C genotype was absent in this cohort (probability of absence of C/C genotype = 1.78×10^−5^) further supporting an association of the T allele with stamina (C/T, 0.32; T/T, 0.68) (Figure 1e). Also, the genotype frequencies among a non-Thoroughbred population known for endurance exercise (*n* = 31, Egyptian Arabian horse) were considerably different to the Thoroughbred population with an excess of T/T (0.90) genotypes (Figure 1f). Furthermore, among a sample of *n* = 35 Quarter Horses, a breed known for short distance racing and activities requiring short bursts of speed, there was an excess of C alleles (0.90; C/C, 0.83; C/T, 0.14; T/T, 0.03). Together these findings indicate that the C/C genotype is particularly suited to fast, shorter distance racing and the T/T genotype confers stamina.

These data indicate that genotypic information at this locus may have practical applications in the Thoroughbred horse racing and breeding industry. To evaluate this further, we investigated two-year-old racing form for *n* = 142 horses-in-training with the same trainer during 2007 and 2008 (*n* = 63, 2007; *n* = 79, 2008) (Table 3). For each parameter of racing success, C/C and C/T genotypes were more successful two-year-old racehorses than T/T animals (Table 3). In terms of earnings, the greatest returns on training investment were for animals that were C/C or C/T; on average these horses earned 5.5-fold more than T/T horses. Even when individuals that had won > Sterling£100,000 (US$165,000) were excluded, on average C/C individuals earned 1.6-fold more than T/T individuals. The bulk of keeping and training expenses are not returned in prize money (72% Ireland, 78% Great Britain for horses that have run in at least one race) [International Federation of Horseracing Authorities, www.horseracingintfed.com]; therefore, employing a strategy to train and race only C/C and C/T individuals as two-year-olds may be beneficial.

**Table 3:** Parameters of two-year-old racing (Ireland and Great Britain) success for *n* = 142 horses-in-training with the same trainer during 2007 and 2008.

	*n*	no. runners	no. winners	total no. races	total no. races won	% runners	% winners to runners	% wins to runners	% winners to total	% wins to runs	mean no. races per runner	total earnings (£)	mean earnings (£)	mean earnings excl. earners >£100k	no. earners >£100k
**Two-year-old horses-in-training**															
CC	40	21	11	87	17	52.5	52.4	81.0	27.5	19.5	4.1	511114	20440	8203	1
CT	67	32	18	115	26	47.8	56.3	81.3	26.9	22.6	3.6	1801103	36968	4925	5
TT	35	13	6	40	6	37.1	46.2	46.2	17.1	15.0	3.1	87461	5175	5175	0
**Two-year-old horses-in-training**															
CC/CT	107	53	29	202	43	49.5	54.7	81.1	27.1	21.3	3.8	2312217	28704	6564	6
TT	35	13	6	40	6	37.1	46.2	46.2	17.1	15.0	3.1	87461	5175	5175	0
**Two-year-old horses-in-training (half-sibs)**															
CT	22	12	9	46	18	54.5	75.0	150.0	40.9	39.1	3.8	1620087	73640	-	6
TT	19	9	5	23	5	47.4	55.6	55.6	26.3	21.7	2.6	67864	3572	-	0

The half-sib two-year-old horses-in-training share a sire.

To eliminate potential confounding effects of shared sires, we also investigated the racing successes of 41 half-sibs (progeny of a single sire) [C/T, *n* = 22; T/T, *n* = 19] (Table 3) that were trained by the same trainer as two-year-olds and found a significant genotype association with racing performance (Pearson's chi-square test: χ^2^ = 7.235; df = 1; *P* = 0.0071); five of the progeny were two-year-old Group race winners and all displayed the C/T genotype.

Similar to human athletes, sprint racing Thoroughbreds are generally more compact and muscular than horses suited to longer distance races. Therefore, to investigate whether *MSTN* genotypes influence body mass we used mass (kg) and height at withers (cm) measurements that were taken during two two-year-old racing seasons for *n* = 97 (*n* = 37 males, *n* = 60 females) horses-in-training with the same trainer. Mass to height ratio displayed a significant (*P* = 0.0147) relationship with g.66493737C>T genotype (2.94 kg/cm, C/C; 2.88 kg/cm, C/T; and 2.83 kg/cm, T/T). This association became stronger when males were considered independently (*P* = 0.0025) of females (*P* = 0.2272) [Table S6]. On average C/C males had 6.7% (*i.e.* 3.033 kg/cm versus 2.843 kg/cm) greater mass per cm than T/T males.

The SNP described here may be used to make predictions about the genetic potential of a horse. While our results do not preclude the functional variant being located in a neighboring gene there are no other plausible candidates within 2 Mb upstream or downstream of the equine *MSTN* gene. In addition, the previous observations demonstrating association with performance in canine athletes point to *MSTN* as a causative gene. Functional genomics studies will inform whether the mutation described here, or its haplotypic background, has an impact on *MSTN* gene expression.

In summary, these findings clearly indicate an opportunity to inform breeding, selection, training and racing decisions through the integration of *MSTN* genotypic information. In all instances, this information will empower Thoroughbred breeders, owners and trainers to make decisions that will maximize a horse's genetic potential leading to reduced operating costs and improved returns on investments.

## Methods

### Ethics

This work has been approved by the University College Dublin, Ireland, Animal Research Ethics Committee.

### Study Animals and Cohorts

The highest standard and most valuable elite Flat races are known as Group (Europe and Australasia) or Stakes races (North America). The most prestigious of these races include The Breeders' Cup races (United States), The Kentucky Derby (United States), The Epsom Derby (Great Britain) *etcetera*. In the United Kingdom and Ireland 196 Group races are competed annually (43 Group 1, 50 Group 2 and 103 Group 3). After Group races, Listed races are the next highest grade of race. To minimize confounding effects of racing over obstacles only horses with performance records in Flat races were considered for inclusion in the principal study cohorts. Horses were categorized based on retrospective racecourse performance records as “Thoroughbred-elite” (TBE) or “Thoroughbred-other” (TBO). Elite Thoroughbreds were Flat racehorses that had won at least one Group race. Other Thoroughbreds were those that had never won a Flat race or had a handicap rating (Racing Post Rating, RPR) <89.

*Association sample:* The International Federation of Horseracing Authorities recognizes five distance categories: Sprint (5–6.5 f, ≤1,300 m), Mile (6.51–9.49 f, 1,301–1,900 m), Intermediate (9.5–10.5 f, 1,901–2,112 m), Long (10.51–13.5 f, 2,114–2,716 m) and Extended (>13.51 f, >2,717 m) races (International Federation of Horseracing Authorities Classifications, www.horseracingintfed.com) [Note: 1 furlong = 1/8 mile = 201.2 meters]. However, for the case-control investigations we compared two cohorts: samples were subdivided into short (≤8 f and ≤7 f) and long (>8 f) distance racing cohorts. To avoid animals with excessive consanguinity (within two generations) and over-representation of popular sires within the pedigrees, a set of Thoroughbred DNA samples (*n* = 148) was selected from a large DNA sample repository (*n*>1,000) collected with informed owners' consent from Thoroughbred training, breeding and sales establishments in Ireland and New Zealand during 1998–2009.

*Replication samples:* To validate the findings, a replication sample of *n* = 62 unrelated elite (Group and Listed race winners) Thoroughbreds was re-sampled from the original repository and supplemented with additional samples collected following the original analyses and genotyped for the g.66493737C>T SNP (Replication sample I).

To minimize non-genetic influences on performance we further validated the findings by genotyping elite (Group and Listed race winning) racehorse samples (*n* = 39) selected from a repository of DNA samples (*n* = 419) from horses trained by the same trainer in Ireland during 2004–2008. This sample had some sharing of relatives, accounted for in the analyses (Replication sample II).

A subset (*n* = 142) of this repository was evaluated for genotypic trends with parameters of racecourse success in two-year-old racehorses. Race records were derived from three sources: European race records, The Racing Post on-line database (www.racingpost.co.uk); Australasian and South East Asian race records, Arion Pedigrees (www.arion.co.nz); and North American race records, Pedigree Online Thoroughbred database (www.pedigreequery.com).

### DNA Extraction, Resequencing, and Genotyping

Genomic DNA was extracted from either fresh whole blood or hair samples using a modified version of a standard phenol/chloroform method [10]. Thirteen pairs of overlapping PCR primers were designed to cover the entire *MSTN* genomic sequence using the PCR Suite extension to the Primer3 web-based primer design tool [11], [12] [Table S1]. Twenty-four unrelated Thoroughbred DNA samples were included in a re-sequencing panel to identify Thoroughbred-specific sequence variants. As such this study was powered to detect 95% of SNPs with MAF>0.05 in the Thoroughbred population [13]. Bidirectional DNA sequencing of PCR products was outsourced to Macrogen Inc. (Seoul, Korea) and carried out using AB 3730xl sequencers (Applied Biosystems, Foster City, CA). Sequence variants were detected by visual examination of sequences following alignment using Consed version 19.0 (090206) [14]. Genotyping was carried out using Sequenom (San Diego, USA) iPlex technology at Sequenom facilities in San Diego, USA (Association samples) and Hamburg, Germany (Replication samples).

### Statistical Analyses

All statistical analyses, including tests of association were performed using PLINK Version 1.05 (http://pngu.mgh.harvard.edu/purcell/plink/) [15], [16]. Quality control analyses included computation of sample allele frequency, percent missing genotypes and deviation from Hardy-Weinberg equilibrium. The series of case-control association tests were performed for two loci (g.66493737C>T and g.66494218A>C). Statistical significance was assessed using the Cochran-Armitage test for trend and an unconditioned genotypic model. Odds ratios and 95% CIs were calculated for the two most significant associations. The linear regression model was used to evaluate quantitative trait association at locus g.66493737C>T using the phenotypes: best race distance and kg/cm ratio.

## Supporting Information

Table S1 Overlapping primer pairs and identified SNPs.
(0.04 MB DOC)

Table S2 *MSTN* SNPs and flanking sequences for genotyping assay design.
(0.03 MB DOC)

Table S3 Genotyping results for *MSTN* SNPs.
(0.03 MB DOC)

Table S4 Population summary including details of retrospective racecourse success for each cohort. RPR = racing post handicap rating. Gr = group race.
(0.03 MB DOC)

Table S5 Hardy-Weinberg equilibrium test results for locus g.66493737C>T.
(0.03 MB DOC)

Table S6 Quantitative association test results for g.66493737C>T with kg/cm ratio as phenotype.
(0.04 MB DOC)
