# Correction: A Sequence Polymorphism in *MSTN* Predicts Sprinting Ability and Racing Stamina in Thoroughbred Horses

**DOI:** 10.1371/annotation/de9e11b9-eb92-4ee5-a56a-908e06d1ed6c

**Published:** 2010-01-26

**Authors:** Emmeline W. Hill, Jingjing Gu, Suzanne S. Eivers, Rita G. Fonseca, Beatrice A. McGivney, Preethi Govindarajan, Nick Orr, Lisa M. Katz, David E. MacHugh

The last author's name was incomplete. The correct name is: David E. MacHugh. 

