# Supplementary material for: A Sequence Polymorphism in MSTN Predicts Sprinting Ability and Racing Stamina in Thoroughbred Horses
Source: PLoS One. 2010 Jan 20;5(1):e8645. doi: 10.1371/journal.pone.0008645 (PMC2808334; doi:10.1371/journal.pone.0008645)
Supplement: Table S1 — Overlapping primer pairs and identified SNPs. (0.04 MB DOC) [file pone.0008645.s001.doc]

**Table S1:** Overlapping primer pairs and identified SNPs

| **Amplicon** | **Primer sequences (5')** | **Primer sequences (3')** | **PCR product size (bp)** | **Chr location (EquCab2.0)** | **No. of SNPs** |
| --- | --- | --- | --- | --- | --- |
| MSTN_1 | ATAAATGCAATTGTCTCAAAGTC | CCATATGCAAGTTTCCATTCC | 399 | chr18:66489320+66489718 | - |
| MSTN_2 | TCAGCCATTCAGCCTATTTG | ACGGTTGGCATTTAACCATC | 422 | chr18:66489629+66490050 | - |
| MSTN_3 | GGAGACTTGCTTTCATTTACCTG | GAAGCTTTTGGATGGGATTG | 552 | chr18:66489914+66490465 | - |
| MSTN_4 | CTCTGGGGTTTGCTTGGTG | ACCTAGGGAATGGAGGATGG | 695 | chr18:66490336+66491030 | - |
| MSTN_5 | GAAGAGGAGGGAGGGAAGAG | TTCAGTCTTCATGTGGTCTTGG | 762 | chr18:66490908+66491669 | - |
| MSTN_7 | AAGGTATTGTCATCTGCTTGG | CCAAGACCAGGAGAAGATGG | 783 | chr18:66491846+66492628 | - |
| MSTN_8 | GCTTGTTAGCATAGGAAACTGG | CTGAGACCCGTCAAGACTCC | 376 | chr18:66492499+66492874 | - |
| MSTN_10 | TGAAGGAATGAACTGTGGATG | GTCTGCGATCCTGCTTTACC | 580 | chr18:66493261+66493840 | 5 |
| MSTN_11 | TTTTGAAACTGTTGTGTCCTG | TCATAATTGCGTTTGGTTGC | 674 | chr18:66493779+66494452 | 1 |
| MSTN_12 | GCAAATGCTCAAATGACCTAAAC | TGTGCTGATTCTTGCTGGTC | 799 | chr18:66494344+66495142 | - |
| MSTN_13 | TGAAGATTTAGTGTTTTGTCTCC | CGAGATTCATTGTGGAGCAG | 382 | chr18:66495028+66495409 | - |
| MSTN_14 | GAGACAACTTGCCACACCAG | TGCCCTGGTAATAACAATGAAG | 786 | chr18:66495287+66496072 | - |
