# Supplementary material for: A Sequence Polymorphism in MSTN Predicts Sprinting Ability and Racing Stamina in Thoroughbred Horses
Source: PLoS One. 2010 Jan 20;5(1):e8645. doi: 10.1371/journal.pone.0008645 (PMC2808334; doi:10.1371/journal.pone.0008645)
Supplement: Table S2 — MSTN SNPs and flanking sequences for genotyping assay design. (0.03 MB DOC) [file pone.0008645.s002.doc]

**Table S2:** *MSTN* SNPs and flanking sequences for genotyping assay design

| **EquCab2.0 SNP location** | **Allele 1** | **Allele 2** | **ASSAY WITH 200BP FLANK** | **Substitution type** |
| --- | --- | --- | --- | --- |
| 66493525 | T | G | TGGAGGAACATCCACTTAGAATTCTTTGGGAATCTGAGTAGTTACACTTACTGAGCAGCTGTACCAATCAGTCTGGAAGAAGGAACCCTTCCCCCAGGCCTGAATTACCTGGGGACAAGACACACTGAGGAACTAACTGAGCCTCGGGAATTAAGAGAAAATATAGTACATCTGTTATGTTTTGGCTTTGGAATAGCCTTT[T/G]AAAGGAACAAAGCTAAGCAAGTAATTAGCACAAAAATTTGAATGTTATATTCAGGCTATCTCAAAAGTTAGAAAATACTGTCTTTAGAGCCAGGCTGTCATTGTGAGCAAAATCACTAGCAATTTCTTTTATTTTGGTTCCCCAAGATTGTTTATAAATAAGGTAAATCTACTCCAGGACTATTTGATAGCAGAGTCATAA | Transversion |
| 66493582 | T | G | GCTGTACCAATCAGTCTGGAAGAAGGAACCCTTCCCCCAGGCCTGAATTACCTGGGGACAAGACACACTGAGGAACTAACTGAGCCTCGGGAATTAAGAGAAAATATAGTACATCTGTTATGTTTTGGCTTTGGAATAGCCTTTTAAAGGAACAAAGCTAAGCAAGTAATTAGCACAAAAATTTGAATGTTATATTCAGGC[T/G]ATCTCAAAAGTTAGAAAATACTGTCTTTAGAGCCAGGCTGTCATTGTGAGCAAAATCACTAGCAATTTCTTTTATTTTGGTTCCCCAAGATTGTTTATAAATAAGGTAAATCTACTCCAGGACTATTTGATAGCAGAGTCATAAAGGAAAATTATTTGGTGCATTATAACCTGATTACTTAATAAGGAGAACAATATTTTG | Transversion |
| **66493737** | **T** | **C** | **AGCTAAGCAAGTAATTAGCACAAAAATTTGAATGTTATATTCAGGCTATCTCAAAAGTTAGAAAATACTGTCTTTAGAGCCAGGCTGTCATTGTGAGCAAAATCACTAGCAATTTCTTTTATTTTGGTTCCCCAAGATTGTTTATAAATAAGGTAAATCTACTCCAGGACTATTTGATAGCAGAGTCATAAAGGAAAATTA[T/C]TTGGTGCATTATAACCTGATTACTTAATAAGGAGAACAATATTTTGAAACTGTTGTGTCCTGTTTAAAGTAGATAAAGCACTGGGTAAAGCAGGATCGCAGACACATGGCACAGAATCTTCCGTGTCATGCCTTCTCTGTGAAGGTGTCTGTCTCCCTTTCCTTGAGTGTAGTTATGAACTGACTGCAAAAAGAATATATG** | **Transition** |
| 66493745 | A | G | AAGTAATTAGCACAAAAATTTGAATGTTATATTCAGGCTATCTCAAAAGTTAGAAAATACTGTCTTTAGAGCCAGGCTGTCATTGTGAGCAAAATCACTAGCAATTTCTTTTATTTTGGTTCCCCAAGATTGTTTATAAATAAGGTAAATCTACTCCAGGACTATTTGATAGCAGAGTCATAAAGGAAAATTATTTGGTGC[A/G]TTATAACCTGATTACTTAATAAGGAGAACAATATTTTGAAACTGTTGTGTCCTGTTTAAAGTAGATAAAGCACTGGGTAAAGCAGGATCGCAGACACATGGCACAGAATCTTCCGTGTCATGCCTTCTCTGTGAAGGTGTCTGTCTCCCTTTCCTTGAGTGTAGTTATGAACTGACTGCAAAAAGAATATATGAAATATAT | Transition |
| 66493775 | A | G | ATTCAGGCTATCTCAAAAGTTAGAAAATACTGTCTTTAGAGCCAGGCTGTCATTGTGAGCAAAATCACTAGCAATTTCTTTTATTTTGGTTCCCCAAGATTGTTTATAAATAAGGTAAATCTACTCCAGGACTATTTGATAGCAGAGTCATAAAGGAAAATTATTTGGTGCATTATAACCTGATTACTTAATAAGGAGAAC[A/G]ATATTTTGAAACTGTTGTGTCCTGTTTAAAGTAGATAAAGCACTGGGTAAAGCAGGATCGCAGACACATGGCACAGAATCTTCCGTGTCATGCCTTCTCTGTGAAGGTGTCTGTCTCCCTTTCCTTGAGTGTAGTTATGAACTGACTGCAAAAAGAATATATGAAATATATTATCTTTCAGAAGCAATATAGATACTACAG | Transition |
| 66494218 | A | C | AGGAGATTATTAAGCAATGTGCCTGCCTGGAAATGTGCACCCCGGGTGCTCTCAACAATAGTACTATGGTCAAGGTGTAAGCAGGACTCTGAGCTATAACCTCTTTGATTAAAATGTTTATTTATTAGGCATTTTATGATAATTAGCTCATGATTATCATTATGCTATGTTTACTTCATCATTTTTCTTACTAATACATTA[A/C]ATTTTAAAAAATATTTTTCCTAATCTCCAGGGGAATAACTTTCAAAATCTAATATGTTAATTTGTGAAGAACATAAAAACACTATGAGAAATAGTTTTGAGTAACAGAAGTCATTTTGGTGTTCAGCAAATGCTCAAATGACCTAAACGTCTACAAATTTCTTCCTTCTCTATTATTAGTGAAAAAAACTTGTTATTATAA | Transversion |
