# Supplementary material for: A Sequence Polymorphism in MSTN Predicts Sprinting Ability and Racing Stamina in Thoroughbred Horses
Source: PLoS One. 2010 Jan 20;5(1):e8645. doi: 10.1371/journal.pone.0008645 (PMC2808334; doi:10.1371/journal.pone.0008645)
Supplement: Table S3 — Genotyping results for MSTN SNPs. (0.03 MB DOC) [file pone.0008645.s003.doc]

**Table S3:** Genotyping results for *MSTN* SNPs

| **Assay (SNP_ID)** | **Coverage** | **NA.** | **Total** | **nallele** | **COMMON** | | **HET** | **RARE** | **p** | **q** | **F_p** | **F_q (MAF)** | **n** |
| --- | --- | --- | --- | --- | --- | --- | --- | --- | --- | --- | --- | --- | --- |
| MSTN_66493525 | 96.67% | 5 | 145 | 2 | 138 | 5 | | 2 | 281 | 9 | 0.969 | 0.031 | 290 |
| MSTN_66493582 | 92% | 12 | 138 | 2 | 135 | 3 | | 0 | 273 | 3 | 0.989 | 0.011 | 276 |
| MSTN_66493737 | 93.33% | 10 | 140 | 2 | 42 | 75 | | 23 | 159 | 121 | 0.568 | 0.432 | 280 |
| MSTN_66493745 | 97.33% | 4 | 146 | 2 | 139 | 6 | | 1 | 284 | 8 | 0.973 | 0.027 | 292 |
| MSTN_66493775 | 96.67% | 5 | 145 | 2 | 139 | 5 | | 1 | 283 | 7 | 0.976 | 0.024 | 290 |
| MSTN_66494218 | 91.33% | 13 | 137 | 2 | 59 | 67 | | 11 | 185 | 89 | 0.675 | 0.325 | 274 |
