# Supplementary material for: A Sequence Polymorphism in MSTN Predicts Sprinting Ability and Racing Stamina in Thoroughbred Horses
Source: PLoS One. 2010 Jan 20;5(1):e8645. doi: 10.1371/journal.pone.0008645 (PMC2808334; doi:10.1371/journal.pone.0008645)
Supplement: Table S4 — Population summary including details of retrospective racecourse success for each cohort. RPR = racing post handicap rating. Gr = group race. (0.03 MB DOC) [file pone.0008645.s004.doc]

**Table S4:** Population summary including details of retrospective racecourse success for each cohort. RPR = Racing Post Handicap Rating. Gr = Group race.

|  | ***n*** | **No. sires** | **No. males** | **No. females** | **Mean RPR** | **Range RPR** | **Total no. races** | **Mean no. races** | **No. races won** | **No. Gr races won** | **No. Gr 1 races won** | **Mean no. Gr races won** |
| --- | --- | --- | --- | --- | --- | --- | --- | --- | --- | --- | --- | --- |
| **TBE** | 86 | 86 | 37 | 49 | 115 | 87-134 | 1170 | 13.8 | 425 | 215 | 91 | 2.5 |
| **TBE > 8 f** | 35 | 35 | 12 | 23 | 119 | 107-134 | - | - | - | 89 | 42 | - |
| **TBE ≤ 8 f** | 51 | 51 | 25 | 26 | 114 | 87-129 | - | - | - | 129 | 49 | - |
| **TBE ≤ 7 f** | 43 | 43 | 20 | 23 | 113 | 87-129 | - | - | - | 76 | 23 | - |
| **TBO** | 62 | 62 | 22 | 40 | 59 | 21 - 89 | 537 | 8.7 | 15 | 0 | 0 | 0 |
