# Supplementary material for: A Sequence Polymorphism in MSTN Predicts Sprinting Ability and Racing Stamina in Thoroughbred Horses
Source: PLoS One. 2010 Jan 20;5(1):e8645. doi: 10.1371/journal.pone.0008645 (PMC2808334; doi:10.1371/journal.pone.0008645)
Supplement: Table S5 — Hardy-Weinberg equilibrium test results for locus g.66493737C>T. (0.03 MB DOC) [file pone.0008645.s005.doc]

**Table S5 Hardy-Weinberg equilibrium test results for locus g.66493737C>T.**

| **TEST** | **A1** | **A2** | **GENO** | **O(HET)** | **E(HET)** | **P** |
| --- | --- | --- | --- | --- | --- | --- |
| ALL | T | C | 23/75/41 | 0.5396 | 0.4916 | 0.3022 |
| TBE <7 f | T | C | 3/16/20 | 0.4103 | 0.4050 | 1 |
| TBE >8 f | T | C | 9/23/0 | 0.7188 | 0.4604 | 0.0018 |
