# Supplementary material for: A Sequence Polymorphism in MSTN Predicts Sprinting Ability and Racing Stamina in Thoroughbred Horses
Source: PLoS One. 2010 Jan 20;5(1):e8645. doi: 10.1371/journal.pone.0008645 (PMC2808334; doi:10.1371/journal.pone.0008645)
Supplement: Table S6 — Quantitative association test results for g.66493737C>T with kg/cm ratio as phenotype. (0.04 MB DOC) [file pone.0008645.s006.doc]

**Table S6** Quantitative association test results for g.66493737C>T with kg/cm ratio as phenotype

|  |  | **Quantitative association test results** | | | |  | **Kg/cm means** | | |  |
| --- | --- | --- | --- | --- | --- | --- | --- | --- | --- | --- |
|  | ***n*** | ***BETA*** | ***SE*** | ***R2*** | ***T*** | ***P*** | **GENO** | **C/C** | **T/C** | **T/T** |
| **Two-year- olds in-training** | 97 | -0.05671 | 0.02282 | 0.06104 | -2.485 | 0.015 | **COUNTS** | 29 | 47 | 21 |
|  |  |  |  |  |  |  | **FREQ** | 0.299 | 0.485 | 0.217 |
|  |  |  |  |  |  |  | **MEAN** | 2.939 | 2.875 | 2.826 |
|  |  |  |  |  |  |  | **SD** | 0.155 | 0.162 | 0.168 |
| **Males only** | 37 | -0.09575 | 0.02941 | 0.2325 | -3.256 | 0.003 | **COUNTS** | 10 | 18 | 9 |
|  |  |  |  |  |  |  | **FREQ** | 0.270 | 0.487 | 0.243 |
|  |  |  |  |  |  |  | **MEAN** | 3.033 | 2.918 | 2.843 |
|  |  |  |  |  |  |  | **SD** | 0.169 | 0.101 | 0.134 |
| **Females only** | 60 | -0.03773 | 0.03091 | 0.02505 | -1.221 | 0.227 | **COUNTS** | 19 | 29 | 12 |
|  |  |  |  |  |  |  | **FREQ** | 0.317 | 0.483 | 0.200 |
|  |  |  |  |  |  |  | **MEAN** | 2.889 | 2.848 | 2.814 |
|  |  |  |  |  |  |  | **SD** | 0.124 | 0.187 | 0.194 |
